# Predictors of measles vaccination coverage among children 6–59 months of age in the Democratic Republic of the Congo

**DOI:** 10.1016/j.vaccine.2017.11.049

**Published:** 2018-01-25

**Authors:** Hayley R. Ashbaugh, Nicole A. Hoff, Reena H. Doshi, Vivian H. Alfonso, Adva Gadoth, Patrick Mukadi, Emile Okitolonda-Wemakoy, Jean Jacques Muyembe-Tamfum, Sue K. Gerber, James D. Cherry, Anne W. Rimoin

**Affiliations:** a Fielding School of Public Health, University of California, Los Angeles, Department of Epidemiology, Los Angeles, CA 90095, United States; b Kinshasa University, School of Medicine, Kinshasa, The Democratic Republic of the Congo; c Kinshasa School of Public Health, Kinshasa, The Democratic Republic of the Congo; d National Institute for Biomedical Research, Kinshasa, The Democratic Republic of the Congo; e Bill and Melinda Gates Foundation, Seattle, WA 98109, United States; f David Geffen School of Medicine at UCLA, Los Angeles, CA 90095, United States

**Keywords:** Vaccination coverage, Measles, Democratic Republic of the Congo, Routine immunization

## Abstract

**Background:**

Measles is a significant contributor to child mortality in the Democratic Republic of the Congo (DRC), despite routine immunization programs and supplementary immunization activities (SIA). Further, national immunization coverage levels may hide disparities among certain groups of children, making effective measles control even more challenging. This study describes measles vaccination coverage and reporting methods and identifies predictors of vaccination among children participating in the 2013–2014 DRC Demographic and Health Survey (DHS).

**Methods:**

We examined vaccination coverage of 6947 children aged 6–59 months. A multivariate logistic regression model was used to identify predictors of vaccination among children reporting vaccination via dated card in order to identify least reached children. We also assessed spatial distribution of vaccination report type by rural versus urban residence.

**Results:**

Urban children with educated mothers were more likely to be vaccinated (OR = 4.1, 95% CI: 1.6, 10.7) versus children of mothers with no education, as were children in wealthier rural families (OR = 2.9, 95% CI: 1.9, 4.4). At the provincial level, urban areas more frequently reported vaccination via dated card than rural areas.

**Conclusions:**

Results indicate that, while the overall coverage level of 70% is too low, socioeconomic and geographic disparities also exist which could make some children even less likely to be vaccinated. Dated records of measles vaccination must be increased, and groups of children with the greatest need should be targeted. As access to routine vaccination services is limited in DRC, identifying and targeting under-reached children should be a strategic means of increasing country-wide effective measles control.

## Highlights

• DRC’s overall measles vaccination coverage level of 70% is too low to halt the spread of measles.
• Socioeconomic variables and residence are associated with vaccination coverage disparities.
• Vaccination coverage and data quality are linked, and as such, dated records must be increased.

## 1 Introduction

Measles is a significant contributor to child mortality, with 114,900 measles deaths reported worldwide in 2014 [1]. Although the World Health Organization (WHO) African Region has committed to measles elimination by 2020 as part of the Global Vaccine Action Plan (GVAP) [2], a number of large outbreaks have recently occurred in sub-Saharan Africa. In the Democratic Republic of the Congo (DRC), the 2010–2013 measles epidemic persisted in light of emergency vaccination campaigns, causing 140,000 cases and 3000 deaths [3], mostly impacting children less than five years of age [4].

Measles vaccination is considered to be one of the most cost-effective public health interventions [5]. Due to the highly contagious nature of the disease, 89–94% of the population must be immunized in order to halt measles transmission [6], [7]. Unvaccinated individuals and vaccinated individuals who fail to mount an immune response are susceptible to measles [8], and the secondary attack rate in families is approximately 90% [9]. Because control and elimination require extremely high levels of vaccination coverage, planning and implementing an effective measles immunization program is critical [7].

New WHO guidance states that all countries should include a second routine dose of measles vaccine (measles containing vaccine 2 (MCV2)), regardless of national coverage level of the first dose (measles containing vaccine 1 (MCV1)) [10], [11]. Measles vaccine effectiveness is 84% when administered at 9 months of age and 93% when administered to children older than 12 months [12]. Measles-endemic areas such as DRC should provide routine vaccinations to children at 9 months and 12–15 months of age, with MCV2 given to protect children not developing immunity after MCV1 [7]. To date, DRC has not implemented this recommendation and is considered “significantly off-track” to meet measles elimination goals by 2020 [13]. In 2015, MCV1 coverage in the African Region was 74%, while MCV2 coverage was only 18% [14]. DRC reported 79% national coverage for the first dose of measles vaccination in 2015, and in 2016, the reported incidence of confirmed measles cases was 6.0 per 100,000, more than double the WHO African Region incidence of 2.7 per 100,000 [11].

Because significant progress in national vaccination coverage levels requires high quality vaccination reporting, both improved reporting of measles vaccination coverage and a tailored and targeted approach are required to effectively reach DRC’s diverse and geographically dispersed population. This study reports measles vaccination coverage, identifies predictors of vaccination, and describes disparities in vaccine documentation by geographic area among children participating in the 2013–2014 DRC Demographic and Health Survey (DHS).

## 2 Methods

The 2013–2014 Demographic and Health Survey (DHS) took place from November 2013 to February 2014, and is a nationally representative survey based on a stratified two-stage cluster design, with the first stage consisting of Enumeration Area (EA) formation and the second of sampling households from each EA [15], [16], [17], [18]. In the first stage, a stratified sample of geographic locations, or clusters (n = 540) were selected with proportional probability according to size. Complete listings of households were created within each cluster, and households were selected with equal probability (n = 9000). Within these households, 18,827 women ages 15–49 and 8656 men ages 15–59 in 50% of selected households were interviewed. Children 6–59 months of age in households from which men were selected were eligible to participate.

Vaccination was reported in three ways. At the time of interview, if mothers possessed a vaccination card provided by a health care worker (HCW) indicating the date the child was vaccinated for measles, this was considered a “dated card” report. If mothers possessed a vaccination card that was marked to indicate measles vaccine was administered, but lacked a date, this was considered a “marked card” report. Finally, if mothers either did not have a card or could not provide a card at the time of interview, yet reported the child had been vaccinated for measles, this was considered a “maternal recall” report.

### 2.1 Statistical analysis

DHS sampling weights were included to account for population sampling methods. There were 7350 children 6–59 months with vaccination data available and 6947 children with all covariates of interest (5.5% missing). Wald chi-square analyses were performed on the weighted sample to assess the sociodemographic differences by vaccination report. Due to increased reliability of recorded data, primary statistical analyses were limited to vaccinated reports via dated card versus the unvaccinated (n = 2830). Potential predictors of vaccination were assessed via univariate and multivariate logistic regression. To incorporate variables capturing health disparities within our sample, we examined social determinants of health [19], [20]. We dichotomized key variables to compare the most disadvantaged to the rest of the population. Specifically, we compared the poorest to all others in our sample, those severely (chronically) malnourished (defined by WHO standards [17]) versus all others, and mothers having no versus any education. Additionally, the United Nations Economic Commission for Africa (UNECA) and the UN Office for the Coordination of Humanitarian Affairs (OCHA) identified the five (old) provinces with the highest levels of human displacement in 2014, most (98%) of which was conflict-related [21], [22]. We compared those living in these provinces (Katanga, Maniema, Nord-kivu, Orientale, and Sud-kivu) versus those living elsewhere. As a sensitivity analysis, the regression model was rerun for children reporting vaccination via maternal recall versus the unvaccinated (n = 6048).

To assess spatial distribution of vaccination report type, maps by (new) province were created. Because 16% of vaccinated children in the overall sample possessed dated cards, we examined whether provinces had levels at or above 16%, between >0 and 15%, or if they had no dated card reports (0%).

The survey was completed on paper questionnaires, and all data were double entered to an electronic format via the Census and Survey Processing System (U.S. Census Bureau, ICF Macro) and compared for accuracy. All analyses were performed using SAS software, Version 9.4 (SAS Institute, Cary, NC), and maps were generated using ArcGIS software version 9.3 (ESRI, Redlands, CA). Ethical approval was obtained at UCLA Fielding School of Public Health, the Kinshasa School of Public Health and the Centers for Disease Control and Prevention. As children were younger than the standard age of assent, the parent or guardian of participating children provided consent on the child’s behalf.

## 3 Results

There were 2058 (30%) children unvaccinated and 4889 (70%) vaccinated for measles (*data not shown*). Among those reporting vaccination, 773 (16%) reported via dated card, 3990 (82%) via maternal recall, and 126 (3%) via marked card. Vaccination report type varied by age, with older children more likely to report vaccination via maternal recall, and also varied by residence, wealth index, and province (Table 1). Among children with recorded date of vaccination, 101/773 (13%) were vaccinated at less than 9 months of age, 567/773 (73%) were vaccinated between 9 and <12 months of age, and 105/773 (14%) were vaccinated at 12 months of age or greater (*data not shown*).

**Table 1:** Vaccination by basic demographics among children 6–59 months of age (weighted).

Variable	n	Dated card	%	Marked card	%	Maternal recall	%	Unvaccinated	%	p-value^a^
*Age (months)*										
6–11	826	62	8	8	1	156	19	600	73	<.0001
12–23	1578	239	15	45	3	788	50	506	32	
24–35	1595	219	14	31	2	1005	63	340	21	
36–47	1492	123	8	27	2	1007	67	335	22	
48–59	1457	130	9	15	1	1035	71	277	19	
*Sex*										
Male	3467	371	11	62	2	2012	58	1022	29	0.7951
Female	3478	401	12	64	2	1978	57	1035	30	
*Residence*										
Urban	2119	346	16	37	2	1236	58	500	24	<.0001
Rural	4829	427	9	90	2	2754	57	1558	32	
*Wealth Index*^b^										
Poorest	1544	77	5	35	2	844	55	588	38	<.0001
Poorer	1617	147	9	27	2	901	56	542	34	
Middle	1390	164	12	16	1	796	57	414	30	
Wealthier	1306	171	13	30	2	784	60	321	25	
Wealthiest	1092	214	20	19	2	666	61	193	18	
*Severe stunting*^c^										
Yes	1678	162	10	36	2	995	59	485	29	0.2924
No	5267	611	12	88	2	2995	57	1573	30	
*Conflict area*										
Yes	2621	404	15	51	2	1460	56	742	28	0.0743
No	4326	368	9	75	2	2531	59	1316	30	
*Province*^d^										
Kinshasa	485	105	22	5	1	301	62	74	15	<.0001
Kwango	357	31	9	5	2	233	65	88	25	
Kwilu	567	22	4	22	4	384	68	139	25	
Mai-Ndombe	313	19	6	---	---	210	67	84	27	
Kongo Central	309	39	13	10	3	190	62	70	23	
Equateur	211	55	26	2	1	93	44	61	29	
Mongala	226	0	0	0	0	139	62	86	38	
Nord-Ubangi	106	3	3	---	---	80	75	23	22	
Sud-Ubangi	329	13	4	9	3	175	53	132	40	
Tshuapa	138	2	2	---	---	78	56	58	42	
Kasai	235	21	9	1	0	123	52	90	38	
Kasai-Central	303	57	19	6	2	132	44	108	36	
Kasai-Oriental	279	27	10	13	5	134	48	105	38	
Lomami	339	9	3	2	0	206	61	122	36	
Sankuru	130	1	1	1	0	53	41	76	58	
Haut-Katanga	301	26	9	4	1	197	65	74	25	
Haut-Lomami	165	7	5	2	1	77	47	79	48	
Lualaba	122	12	10	5	4	48	39	57	47	
Tanganyka	137	3	2	---	---	48	35	86	63	
Maniema	235	10	4	---	---	135	57	90	38	
Nord-Kivu	549	178	32	11	2	277	50	83	15	
Bas-Uele	148	6	4	---	---	102	69	40	27	
Haut-Uele	104	7	7	2	2	66	63	29	28	
Ituri	180	9	5	15	8	115	64	41	23	
Tshopo	156	5	3	3	2	81	52	67	43	
Sud-Kivu	522	105	20	8	2	313	60	96	18	
Total observations^e^	6947									

a Wald chi-square test.

b Wealth index is the Demographic and Health Survey composite measure of a household's cumulative living standard. Based on household ownership of previously selected assets, and utilizing principal components analysis, households are placed within one of five quintiles.

c Severe stunting as defined by the NCHS/CDC/WHO international references standard for height/age SD.

d Wald chi-square for province does not include “marked on card” group due to empty cells.

e Unweighted total observations = 7107.

Limiting vaccination reports to children possessing dated card (excluding “maternal recall” and “marked card” reports), univariate analyses (Table 2) revealed that increasing age (by month) was predictive of having received measles vaccination (OR = 1.02, 95% CI: 1.01, 1.03), as was being firstborn, having a mother with at least primary education, being wealthier, living in an urban area, and living in a province experiencing high levels of conflict-related displacement. In the multivariate model limited to urban residence, similar trends were seen, with the exception of a negative association among the most malnourished children (OR = 0.4, 95% CI: 0.2, 0.6), and a greater predictive association among urban children with educated mothers (OR = 4.1, 95% CI: 1.6, 10.7). Limiting observations to rural residence revealed no association with severe chronic malnutrition or maternal education, and increased odds of vaccination for wealthier children (OR = 2.9, 95% CI: 1.9, 4.4) and those in conflict areas (OR = 2.1, 95% CI: 1.3, 3.4).

**Table 2:** Predictors of measles vaccination among children 9–59 months of age (report via dated card only).

Variable	Unadjusted^a^ OR and 95% CI	Adjusted^b^ OR and 95% CI (Urban)	Adjusted^c^ OR and 95% CI (Rural)
Age (months)	**1.02 (1.01, 1.03)**	**1.03 (1.02, 1.04)**	**1.02 (1.01, 1.03)**
*Sex*			
Male	Ref	Ref	Ref
Female	1.1 (0.8, 1.3)	1.0 (0.7, 1.4)	1.2 (0.8, 1.7)
*Chronic malnutrition*^d^			
No	Ref	Ref	Ref
Yes	0.9 (0.6, 1.1)	**0.4 (0.2, 0.6)**	1.2 (0.9, 1.6)
*Birth order*^e^			
Firstborn	Ref	Ref	Ref
Non-firstborn	**0.7 (0.5, 0.9)**	**0.5 (0.3, 0.7)**	0.9 (0.5, 1.5)
*Mother's education*			
No education	Ref	Ref	Ref
Primary or higher	**1.6 (1.0, 2.6)**	**4.1 (1.6, 10.7)**	1.2 (0.8, 2.0)
*Residence*			
Urban	Ref	---	---
Rural	**0.4 (0.3, 0.6)**	---	---
*Wealth Index*			
Poorest	Ref	Ref	Ref
Wealthier	**3.6 (2.6, 5.2)**	1.8 (0.6, 5.3)	**2.9 (1.9, 4.4)**
*Conflict area*			
No	Ref	Ref	Ref
Yes	**1.6 (1.1, 2.3)**	1.3 (0.8, 2.1)	**2.1 (1.3, 3.4)**

Bolding indicates a statistically significant estimate at a significance level of 0.05.

a Univariate analyses, among children with dated measles vaccination card versus unvaccinated (n = 2830).

b Adjusted for all predictors in table, among urban residents (n = 845).

c Adjusted for all predictors in table, among rural residents (n = 1985).

d Chronic malnutrition (according to NCHS/CDC/WHO international references standard for height/age SD).

e Birth order ranges from firstborn to 15th-born.

In sensitivity analyses limiting vaccination to those reporting via maternal recall (versus unvaccinated), univariate analyses (Table 3) revealed trends similar to the main regression model, but with fewer statistically significant estimates (birth order, conflict area) and a general decrease in estimate magnitude. In the multivariate model limited to urban residence, similar trends were seen, but most estimates were not statistically significant. Chronic malnutrition retained its significant negative association, but decreased in magnitude (OR = 0.6, 95% CI: 0.4, 0.8). Limiting observations to rural residence, only increasing age and wealthier children (OR = 1.3, 95% CI: 1.1, 1.7) demonstrated a predictive association with vaccination.

**Table 3:** Predictors of measles vaccination among children 6–59 months of age (report via maternal recall only).

Variable	Unadjusted^a^ OR and 95% CI	Adjusted^b^ OR and 95% CI (Urban)	Adjusted^c^ OR and 95% CI (Rural)
Age (months)	**1.05 (1.0, 1.1)**	**1.1 (1.0, 1.1)**	**1.04 (1.04, 1.05)**
*Sex*			
Male	Ref	Ref	Ref
Female	0.97 (0.8, 1.1)	0.95 (0.7, 1.2)	0.95 (0.8, 1.1)
*Chronic malnutrition*^d^			
No	Ref	Ref	Ref
Yes	1.1 (0.9, 1.3)	**0.6 (0.4, 0.8)**	1.0 (0.8, 1.3)
*Birth order*^e^			
Firstborn	Ref	Ref	Ref
Non-firstborn	1.1 (0.9, 1.3)	0.9 (0.6, 1.2)	1.1 (0.9, 1.4)
*Mother's education*			
No education	Ref	Ref	Ref
Primary or higher	**1.4 (1.1, 1.7)**	1.7 (0.9, 3.4)	1.3 (0.99, 1.7)
*Residence*			
Urban	Ref	---	---
Rural	**0.7 (0.6, 0.9)**	---	---
*Wealth Index*			
Poorest	Ref	Ref	Ref
Wealthier	**1.5 (1.2, 1.8)**	1.3 (0.6, 2.8)	**1.3 (1.1, 1.7)**
*Conflict area*			
No	Ref	Ref	Ref
Yes	1.0 (0.8, 1.3)	1.2 (0.8, 1.8)	1.0 (0.8, 1.4)

a Univariate analyses, among children with maternal recall report versus unvaccinated (n = 6048).

b Adjusted for all predictors in table, among urban residents (n = 1736).

c Adjusted for all predictors in table, among rural residents (n = 4312).

d Chronic malnutrition (according to NCHS/CDC/WHO international references standard for height/age SD).

e Birth order ranges from firstborn to 15th-born.

**Fig. 1:**
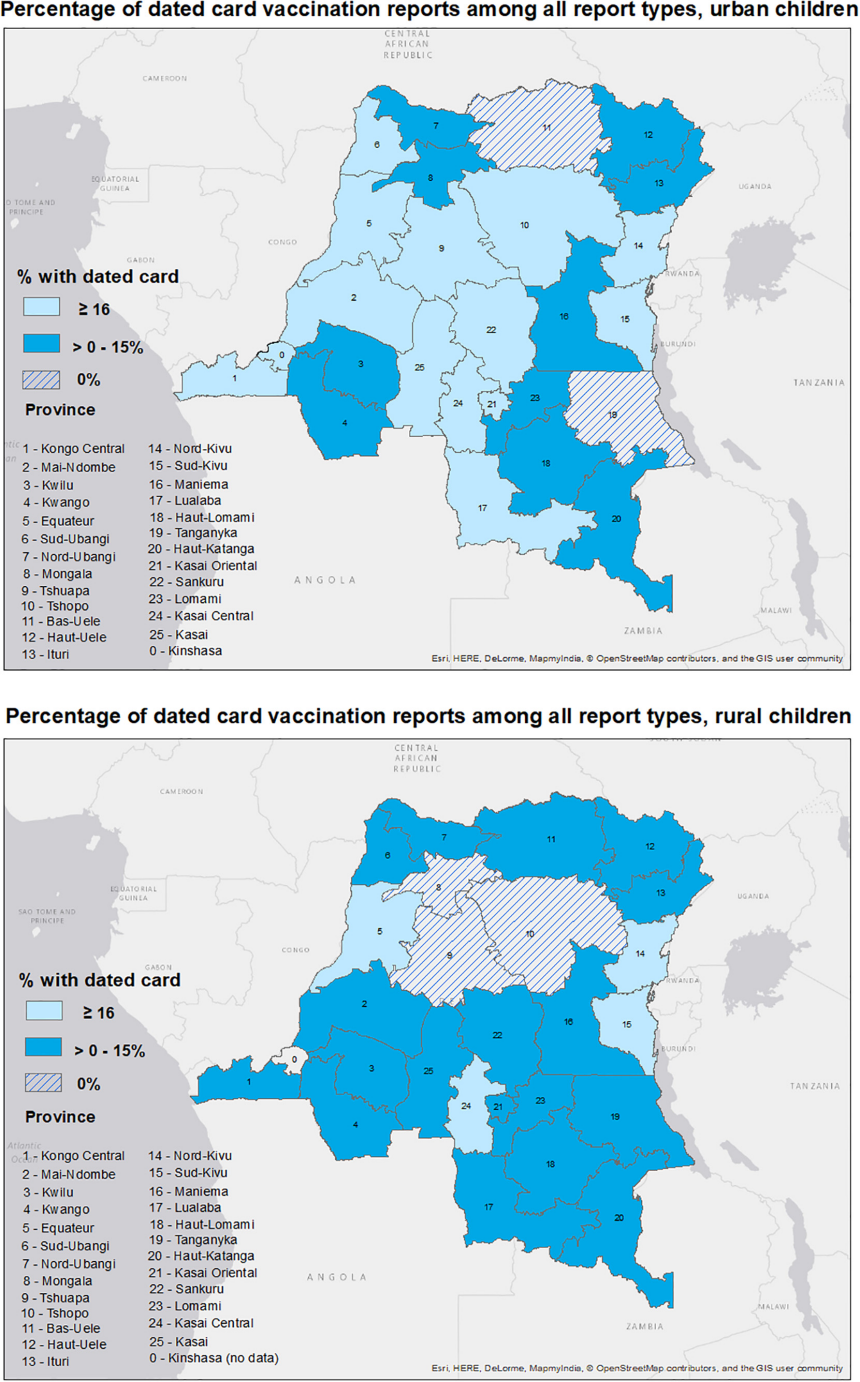
Percentage of children reported vaccination via dated card among all report types, stratified by urban (upper panel) versus rural (lower panel) residence. Among the entire sample, 16% of vaccinated children reported data via dated card.

Mapping the percentage of vaccination reports via dated card at the (new) provincial level revealed different patterns in urban versus rural areas (Fig. 1). As previously mentioned, within the overall sample, 16% of vaccinated children reported immunization via dated card. In urban areas, 14/26 (54%) provinces reported 16% or more reports came from dated vaccination cards, while only 4/25 (16%) provinces in rural areas reported 16% or higher levels of dated card report. Because Kinshasa province has no rural areas designated in the DHS, it is not counted as a rural province when stratifying by residence, and as such, only 25 rural provinces exist.

## 4 Discussion

Reported MCV coverage estimates are too low to effectively halt measles transmission. More troubling, however, is that national or regional reports may mask underlying population disparities, particularly regarding wealth index and urban versus rural residence [23]. Younger children, rural children in the poorest wealth quintile, severely stunted urban children, urban children with uneducated mothers, and non-firstborn urban children were less likely to receive measles vaccination. Documentation of immunization is also inadequate, with rural children less frequently possessing a dated vaccination card compared to urban children at the provincial level.

Vaccination coverage and data quality are linked. As such, progress in measles coverage and schedule adherence cannot be reliably assessed without improvements in data quality. Approximately one third of children under 5 years remain unvaccinated, and of those vaccinated, 83% of reports are via maternal recall. Although information bias from recall reporting could result in either an over- or underestimate of vaccination, recall is generally considered a less reliable source of information than dated vaccination card [24]. Ensuring that children receive vaccinations according to recommended schedules is important as well.

Records of routine immunization should be improved in order to produce more accurate coverage estimates [25], and tracking deviations from the vaccine schedule via dated records is of critical importance as age at vaccination can affect immune response [26]. Particularly in light of the new WHO MCV2 recommendations, dated records are needed to assess vaccine administration for all DRC children. Priority must be given not only to improving vaccination coverage, but also to recording vaccinations in a consistent and accurate manner. An increase in dated, HCW-recorded vaccination data could aid in identifying the greatest areas of need and so more effectively drive immunization programming and policy.

An immunization program targeting the most disadvantaged and difficult to reach children can improve measles coverage and control at a national level, and this requires careful identification of children with the least access to health care. This method has been demonstrated in DRC and elsewhere by WHO’s Reach Every District (RED) component to reach all target populations, including under-served and difficult to reach communities, in efforts to increase access to health services [27], [28]. Further, because predictors of vaccination can affect urban children differently than rural children, residence should be taken into account when identifying children of greatest need. In countries where such population disparities exist, the fastest increases in overall measles vaccination coverage have occurred when coverage is improved among the poorest [29] and most geographically remote [19].

Conflict has been a source of health disparity in affected areas in DRC; as of 2016, there have been over 2.2 million internally displaced people (IDP), primarily due to armed conflict or related reasons [21], [30]. Our findings revealed that residing in a province experiencing displacement due to conflict was predictive of vaccination. One reason for this could be the increased number of aid workers in these regions to provide such services. This is consistent with other recently published studies showing that reactive campaigns in high-risk areas, such as those led by Médecins Sans Frontières (MSF), tend to improve vaccination coverage levels and records [31], [32]. Because conflict has played a major role in the recent history of DRC, further research is warranted to determine how best to incorporate the impact of conflict on health in DRC, and subsequently, how to develop an appropriate programmatic response.

Logistic and infrastructural limitations within the DRC health care delivery system itself also contribute to inadequate vaccination coverage. Clinics may be short-staffed, lack regular vaccine deliveries and may not possess equipment or have consistent electricity/generators needed to adequately maintain the cold chain [33], [34]. Moreover, unreliable cold chain, coupled with an SIA time restriction of five days are problematic, as such limitations may preclude these campaigns effectively reaching children in remote locations [33]. Finally, coverage estimates can be unreliable, with administrative reports differing from supplementary immunization activity (SIA) data and that of non-governmental organizations (NGOs) such as MSF [31], [33], underscoring the need for high quality, reliable data.

Strengths of this study include a large, nationally representative sample, and availability of dated vaccination card and georeferenced data. To address concern of vaccination misclassification, we limited statistical analyses to vaccinations reported via dated card, increasing confidence in vaccination data. Limitations include lack of individual-level data for measles immunization campaigns, resulting in potentially more children vaccinated than our data indicate. Additionally, although the availability of dated vaccination cards strengthened our analysis, a minority of children who reported measles vaccination possessed these cards, and these children differed from children reporting via maternal recall. To mitigate this issue, we performed a sensitivity analysis with the same regression model, limited to children reporting vaccination via maternal recall (versus unvaccinated). Finally, measuring the association of conflict with measles vaccination was challenging, and our assessment of provinces experiencing displacement due to conflict may not have captured the true impact of conflict on vaccination services.

To summarize, results of this study indicate three main areas of need in DRC’s immunization program. Most importantly, there is a need for increased vaccination coverage and better adherence to vaccination schedules. Next, improved data quality is needed to better assess progress in vaccination coverage. Finally, a tailored approach must be developed for DRC that targets the most disadvantaged and underserved children. Incorporating these three components into future vaccination strategies for DRC may be key to bettering national vaccination coverage levels that lead to improved measles control.

## Funding

This work was funded by the Faucett Catalyst Fund, Los Angeles, CA; and the Estimating Population Immunity to Poliovirus in the Democratic Republic of the Congo Grant by the Bill and Melinda Gates Foundation, Seattle, WA [Grant No. OPP1066684].

## Disclaimers

The views expressed are the authors' alone and do not reflect the official policy or position of the Department of the Army, Department of Defense, or the U.S. Government.

## Conflict of interest

None.
